# Supplementary material for: ACE-031, a soluble activin type IIB receptor, increases muscle mass and strength in the common marmoset (Callithrix jacchus)
Source: PLoS One. 2026 Feb 13;21(2):e0342666. doi: 10.1371/journal.pone.0342666 (PMC12904423; doi:10.1371/journal.pone.0342666)
Supplement: Attachment — Submitted filename: Response to Reviewers.docx [file pone.0342666.s006.docx]

DATE: January 5, 2025

RE: Revision of Manuscript #PONE-D-25-54403R1

**Response to Reviewer Comments (PONE-D-25-54403R1)**

The Authors want to thank the Editor and Expert Reviewers for the thoughtful and thorough review of our revised submitted manuscript “*ACE-031, a Soluble Activin Type IIB Receptor, Increases Muscle Mass and Strength in the Common Marmoset (Callithrix jacchus).”* A point-by-point response to the Reviewer suggestions is presented below.

Reviewer #2: Thank you for the revised manuscript and the detailed responses. The authors have made clear improvements, and many of the earlier concerns are now addressed. The paper reads better, the analyses are more transparent, and the Discussion has more depth. I appreciate the effort put into the revisions. Below are my updated comments.

*Response:* Thank you.

1. Statistical clarity
The authors corrected the statistical approach and re-ran the tests as two-tailed, which is appropriate. This fixes one of the main issues from the previous review. However, the interpretation of the mixed-model results is still a bit difficult for readers who are not familiar with these analyses. In the current form, the Results section may still leave some confusion.
Add 1–2 short sentences explaining, in simple terms, what the significant Time × Treatment interaction means in practice (e.g., lean mass increased over time in the treated group but stayed stable in controls). This will make the results easier to understand.

*Response:* Thank you for this suggestion. We have added a sentence to the Results section to clarify the mixed models results.

2. Muscle function interpretation
The additional explanation on force measurements is helpful. Still, there is a structural–functional gap:
• hypertrophy was measured mainly in the biceps
• force was measured in the EDL
The authors justify this choice scientifically, but a brief reminder in the Discussion that systemic therapies may not produce identical effects in every muscle would help readers understand this limitation.

*Response:* We have added text to the end of the Discussion to address this limitation. Specifically, we added in the rationale for the use of the EDL for functional assessment, and then added the text suggested by the Reviewer regarding the potential that systemic therapies may not similarly affect all muscles.

3. Some figure legends still have small grammatical issues and could be proofread once more.

*Response:* Thank you for this and we apologize for that overcite. The figure legends have been corrected and should not contain any additional grammatical errors.

4. Use consistent terminology for lean mass throughout the paper.

*Response:* Thank you. We have used “lean body mass” throughout the manuscript.

5. Ensure that all figure labels match the panels exactly.

*Response:* We have checked figure labels and the figures to ensure they match.

The manuscript is clearly improved. The authors addressed most of the earlier concerns in a meaningful way, and the scientific content is now stronger and more transparent. A few remaining issues relate to clarity and presentation rather than major flaws.

*Response:* Thank you once again for the additional suggestions and corrections to our manuscript.
