# Supplementary material for: ACE-031, a soluble activin type IIB receptor, increases muscle mass and strength in the common marmoset (Callithrix jacchus)
Source: PLoS One. 2026 Feb 13;21(2):e0342666. doi: 10.1371/journal.pone.0342666 (PMC12904423; doi:10.1371/journal.pone.0342666)
Supplement: Attachment — Submitted filename: Plos One Revisions and Reviewer Comments.docx [file pone.0342666.s005.docx]

DATE: December 14, 2025

RE: Revision of Manuscript #PONE-D-25-54403

**Response to Reviewer Comments (PONE-D-25-54403)**

The Authors want to thank the Editor and Expert Reviewers for the thoughtful and thorough review of our submitted manuscript “*ACE-031, a Soluble Activin Type IIB Receptor, Increases Muscle Mass and Strength in the Common Marmoset (Callithrix jacchus).”* We have carefully considered all the suggestions and revised the manuscript accordingly. We have also addressed the additional requirements required for publication in *PLOS ONE*, which has strengthened our manuscript considerably. A point-by-point response to each Reviewer suggestion is presented below, which we hope sufficiently addressed all Reviewer concerns.

Reviewer #1:

1) This manuscript presents a well-conducted preclinical study investigating the effects of ACE-031, a soluble ActRIIB receptor, on muscle mass and function in the common marmoset, a non-human primate (NHP) model. The study is timely and addresses a critical gap in the field of myostatin/activin pathway inhibition, namely, the translation of promising murine data to a more clinically relevant primate model. The experimental design is robust, incorporating longitudinal body composition analysis, terminal morphometric assessment of muscle fibers, and functional ex vivo contractility measurements. The results clearly demonstrate that ACE-031 treatment increases lean mass, muscle fiber cross-sectional area, and muscle strength in marmosets.

*Response:* Thank you.

2) While the findings are positive and support the therapeutic potential of broad ActRIIB ligand inhibition, the manuscript has significant weaknesses in its statistical reporting, data interpretation, and discussion that must be addressed prior to publication.

*Response:* We have revised the manuscript to address the identified weaknesses in statistical reporting, data interpretation and discussion.

3) The study uses a control group of n=4 and a treatment group of n=8. There is no justification provided for this sample size, and no power calculation is reported. With such a small control group (n=4), the study is severely underpowered. This increases the risk of both Type I and Type II errors. The authors themselves note that one outlier in the control group (Marmoset "A") may have affected the group means and statistical outcomes. A small n amplifies the impact of such outliers.

*Response:* We agree with the Reviewer and concede that this was indeed a limitation of the study. We have included the following paragraph in the Discussion to identify this issue:

“A limitation of this study is that the relatively small sample size, particularly in the control group (n = 4; 2 male, 2 female) compared with the treated group (n = 8; 4 male, 4 female), may have resulted in inadequate statistical power to detect subtle but potentially meaningful treatment effects. This limitation was underscored by a vehicle treated animal that showed an unexpected increase in body weight and muscle mass over the course of the study—an outcome anticipated in the ACE-031 treated group—which likely contributed to variability, limiting the ability to detect group differences. The authors concede that this limitation should be taken into account when interpreting the findings and their generalizability.”

We hope this addition to the Discussion addresses the Reviewer’s concern.

4) The abstract states, "Marmosets administered ACE-031 showed a significant increase in body weight and lean body mass from baseline, while no change was seen in the vehicle-treated controls." However, the statistical analysis in the results (Fig 1A, B) reports a main effect of time, but no main effect of treatment and no significant interaction for body weight. For lean mass, there is a significant interaction but no main effect of treatment. This indicates that while the ACE-031 group increased over its own baseline, the statistical comparison between the treatment and control groups over time is not robust. The phrasing in the abstract is therefore misleading and must be corrected to accurately reflect the statistical findings.

*Response:* We have revised the wording in the Abstract to more accurately reflect the statistical findings.

5) The use of one-tailed t-tests is questionable and requires strong justification. One-tailed tests should only be used when there is a priori, directional hypothesis with no possibility of an effect in the opposite direction. While the hypothesis was for an increase, the use of one-tailed tests doubles the risk of a false positive. The journal's policy on this should be checked, and it is standard practice to use two-tailed tests unless there is an overwhelming justification otherwise. All analyses using one-tailed tests should be re-run with two-tailed tests, or a compelling justification must be provided.

*Response:* The use of the one-tailed t-test was in response to the large body of literature indicating that myostatin and ActRIIB inhibition, either through genetic or pharmacologic strategies, was associated with greater skeletal muscle mass and strength [1-14]. We felt that the one-tailed t-test was justified, as we expected to see a similar response in the NHP marmoset model. However, given the uncertainty of moving from murine models to NHP models, the Reviewers concern, and *PLOS ONE’*s policy, we re-analyzed the data using a two-tailed t-test. The majority of the parameters analyzed by t-test remained unchanged with regards to statistical significance. The one parameter that was changed from significant to not significant was the EDL absolute twitch force: one-tailed t-test p=0.0266; two-tailed t-test p=0.0533. We have revised the manuscript to reflect this change in statistical analysis, including revised text in the Materials and methods and Results sections and revised Figure 3. We thank the Reviewer for this suggestion and hope that the revised statistical analysis strengthens the conclusions of the data.

6) The discussion reads like a positive interpretation of the data without a balanced critical perspective. It fails to adequately address the central paradox of the ACE-031 clinical program: why did a drug that shows efficacy in NHPs (this study) and humans (increased lean mass) fail in Phase II trials for Duchenne Muscular Dystrophy (DMD) due to safety concerns (epistaxis, telangiectasias)?

*Response:* We acknowledge that this is indeed a potential safety concern with this class of drug. We’ve added more to this point in the Discussion:

“Despite the potential of ActRIIB ligand traps to ameliorate muscle wasting, the experience from the ACE-031 clinical program underscores important safety considerations for this drug class. ACE-031 was discontinued in a Phase II trial in Duchenne muscular dystrophy due to vascular-related adverse events, including epistaxis, telangiectasia, and gum bleeding, which were later attributed to inhibition of ligands beyond myostatin and activin A, particularly BMP9[15], which plays a critical role in vascular homeostasis. This discovery highlights that broad inhibition of TGF-β family ligands can produce effects that extend beyond muscle. Thus, while ActRIIB-targeted therapies remain a promising approach to increasing muscle mass and function, the ACE-031 findings emphasize the need for more selective ligand targeting and the inclusion of monitoring of vascular endpoints in future clinical trials. Indeed, RKER-06...

7) The discussion on fiber types is informative but could be more incisive. The authors conclude that the inherent metabolic profile is unaltered, which is a strength. However, they could more directly discuss the potential clinical implications of hypertrophy in both fiber types versus a selective effect.

*Response:* Thank you for the suggestion. We agree that this is an important aspect of our treatment paradigm the deserves a more detailed discussion. The Discussion has been edited to include the following:

“Slow‐twitch fibers maintain oxidative capacity and fatigue resistance, supporting posture and endurance-based tasks, whereas fast‐twitch fibers provide high power output and rapid contractile kinetics needed for short bursts of energy. Therapeutics that induce fiber type transitions may result in functional trade-offs (e.g., resistance to fatigue at the expense of maximal force production, or vice versa). This has been demonstrated in myostatin KO mice where selective increase in type II fibers at the expense of type I is associated with reduced endurance capacity [16] and accelerated fatigue in isolated muscles[1]. Similarly, post-developmental KO of myostatin was associated with a significant reduction in voluntary wheel running and wild-type adult mice administered an AAV expressing the myostatin propeptide to antagonize myostatin activity presented with reduced forced distance running and greater fatigability in isolated muscles [17]. To date, a similar analysis in humans treated with muscle anabolics has not been completed but some insights may be garnered from examples of selective fiber type loss in the context of muscle atrophy. For example, elderly individuals with sarcopenia exhibit a selective atrophy of type II fibers [18-20], which has been shown to be associated with fracture related falls that may be attributed to inability to generate sufficient force needed to overcome loss of balance[18, 20]. In contrast, heart failure patients present with a slow to fast fiber type transition that is associated with severe intolerance to physical exercise and accelerated fatigue during normal activities of daily living [21, 22]. Thus, a muscle anabolic that preserves the pre-treatment fiber type profile is likely to benefit the patient by maintaining energy to perform normal activities of daily living while retaining the force generating capacity to prevent falls and injuries.”

Reviewer #2:

1) This paper studies the effects of ACE-031, a protein that blocks activin receptor type IIB ligands, on muscle growth and strength in common marmosets. The topic is important and relevant, especially for developing new treatments for muscle-wasting diseases. The study is well organized, and the writing is clear. Using a non-human primate model makes the results more meaningful for human application. However, there are some problems with the experimental design, data analysis, and interpretation that should be fixed before the paper can be published.

*Response:* Thank you for the positive words on our manuscript. We have revised the manuscript to address your concerns on the experimental design, data analysis and interpretation.

2) The goal of testing ACE-031 in primates is reasonable, since mouse models don’t always match human biology. Still, the novelty is limited because ACE-031 and other ActRIIB blockers have been studied before. The authors should explain what new information this study adds. For example, what unique features of the marmoset model make these results important? Emphasize publications that show NHP to be superior to rodents when evaluating ACTRIIB ligand traps. E.g., different levels of circulating ligands in rodents vs NHP/humans.

*Response:* Thank you for the suggestion. This was briefly mentioned in the Introduction but is worthwhile to revisit and further expand upon in the Discussion. We have now included the following in the Discussion:

“Activin A levels in monkeys and humans are 3-4 fold higher than mice and rats [23] and myostatin levels in mice are more than 10-fold higher than humans[24] suggesting that myostatin specific treatments that appear promising in mice are not as likely to produce clinically meaningful outcomes in humans. Indeed, although myostatin specific treatments have been shown to be efficacious in a mouse model of muscular dystrophy [2, 3, 25], selective antagonism of myostatin has failed to show meaningful clinical benefit in patients with muscular dystrophies [26-28]. Further, a systematic analysis of the pharmacokinetic and pharmacodynamic profile of a myostatin neutralizing antibody in mice, rats, monkeys and humans suggest that the amount of drug needed to induce comparable increases in muscle mass in monkeys was 20-fold higher than what is needed in mice; indicating substantial species differences in sensitivity to myostatin specific treatments[29]. Thus, the use of NHPs is likely to be a superior predictor of efficacy for treatments targeting myostatin and activin A in humans.”

We hope this added text sufficiently addresses the Reviewer’s concerns.

3) Only 12 animals were used (8 treated, 4 controls). This small sample size reduces the strength of the findings. Also, using one-tailed t-tests is not appropriate here, since the direction of effect was not fully predictable. Use two-tailed tests, include a power calculation, and explain how the small sample size might affect the results.
   *Response:* Your concerns regarding the sample size and the use of a one-tailed t-test were shared with Reviewer 1. We have added the following text to the Discussion regarding the same sizes:

“A limitation of this study is that the relatively small sample size, particularly in the control group (n = 4; 2 male, 2 female) compared with the treated group (n = 8; 4 male, 4 female), may have resulted in inadequate statistical power to detect subtle but potentially meaningful treatment effects. This limitation was underscored by a vehicle treated animal that showed an unexpected increase in body weight and muscle mass over the course of the study—an outcome anticipated in the ACE-031 treated group—which likely contributed to variability, limiting the ability to detect group differences. The authors concede that this limitation should be taken into account when interpreting the findings and their generalizability.”

Regarding the use of a one-tailed t-test, this was in response to the large body of literature indicating that myostatin and ActRIIB inhibition, either through genetic or pharmacologic strategies, was associated with greater skeletal muscle mass and strength [1-14]. We felt that the one-tailed t-test was justified, as we expected to see a similar response in the NHP marmoset model. However, given the uncertainty of moving from murine models to NHP models, the Reviewers concern, and *PLOS ONE’*s policy, we re-analyzed the data using a two-tailed t-test. The majority of the parameters analyzed by t-test remained unchanged with regards to statistical significance. The one parameter that was changed from significant to not significant was the EDL absolute twitch force: one-tailed t-test p=0.0266; two-tailed t-test p=0.0533. We have revised the manuscript to reflect this change in statistical analysis, including revised text in the Materials and methods and Results sections and revised Figure 3. We thank the Reviewer for these suggestions and hope that the revisions strengthen the conclusions of the data.

4) The results show increases in lean mass and body weight, but some effects were not significant when using two-way ANOVA. One animal in the control group gained a lot of weight, which likely affected group averages. Re-analyze the data with and without that animal and discuss how it changes the outcome. Show individual data points in the figures to make variability clear.
   *Response:* We agree with the Reviewer that this one control marmoset contributes to variability in our data. We have performed the statistical analysis as suggested by the Reviewer. Here we provide a comparison of the statistical analysis for body weight and lean body mass with and without that one specific marmoset. As seen in the analysis, the statistical significance does not change when removing the data points associated with this marmoset. For this reason, we feel
it is better to maintain the original analysis that includes this marmoset.

We have also provided here new graphs for the body weight, lean body mass and fat mass that show data points for each individual marmoset.

These graphs become quite busy in this format. It is our preference to utilize the figures contained within our original submission. However, if the Reviewer feels these new graphs better present the variability in the data and allow a better interpretation by readers, we will make the change.

5) The study measured strength in the EDL muscle, but muscle growth was mainly shown in the biceps. This makes it hard to connect the structural and functional results. Explain why EDL was used instead of biceps or include strength data from a muscle that also showed hypertrophy.

*Response:* The use of the EDL for functional measures was based on anatomical and methodological issues. The EDL muscle is a fusiform muscle, in which the muscle fibers run parallel to each other, with no pennation angle, and taper at proximal and distal myotendinous junctions. Surgically, the EDL can be dissected with the proximal and distal tendons intact, leaving the muscle undamaged for subsequent functional assessment. This makes the EDL muscle ideal for functional studies as isometric force is directed in one direction between the connections to the force transducer and muscle stimulation chamber. Also, the volume of the muscle stimulation chamber and the maximum force associated with our available force transducer limited the muscles we could have analyzed ex vivo. In addition, our laboratories [2, 3, 12, 30-32] have reported on the functional responses of the mouse EDL muscles in disease models and in response to myostatin and ActRIIB inhibition. This allows the functional data collected in the EDL muscles from marmosets to be compared to prior functional datasets. For these reasons, the EDL was utilized for functional assessment, and we hope these address the Reviewer’s concerns.

6) The study does not report much about safety. Since ACE-031 has been linked to bleeding problems in past human studies, this part is important. Include data or at least a summary on blood tests, organ changes, or any visible side effects.

*Response:* Your concern was shared by Reviewer #1. We acknowledge that this is indeed a potential safety concern with this class of drug. We’ve modified the header of the first Results paragraph to include safety and tolerability and have included the following statement:

The drug was generally well tolerated, and marmosets showed no adverse effects associated with treatment. Blood samples were collected at several intervals throughout the course of the study to evaluate serum chemistries and all values remained within normal ranges (Table 1). Upon necropsy, no gross safety concerns or morphological abnormalities in tissues or organs were observed.

We’ve also added more to this point in the Discussion:

“Despite the potential of ActRIIB ligand traps to ameliorate muscle wasting, the experience from the ACE-031 clinical program underscores important safety considerations for this drug class. ACE-031 was discontinued in a Phase II trial in Duchenne muscular dystrophy due to vascular-related adverse events, including epistaxis, telangiectasia, and gum bleeding, which were later attributed to inhibition of ligands beyond myostatin and activin A, particularly BMP9, which plays a critical role in vascular homeostasis [15]. This discovery highlights that broad inhibition of TGF-β family ligands can produce effects that extend beyond muscle. Thus, while ActRIIB-targeted therapies remain a promising approach to increasing muscle mass and function, the ACE-031 findings emphasize the need for more selective ligand targeting and the inclusion of monitoring of vascular endpoints in future clinical trials. Indeed, RKER-065…

We also provided data in S1 Table to support that ACE-031 was not associated with abnormal serum chemistry. We hope these data, along with the added text in the Discussion is sufficient to address the Reviewer’s concerns.

7) The Discussion repeats a lot from previous studies and doesn’t say much about what these specific results mean. It would help to explain the possible biological reasons for the observed effects, such as which muscle pathways might be involved. Add more explanation about how ACE-031 might cause muscle growth and strength increases and compare how this may differ from mice or humans.

*Response:* The goal of this study was to extend the data obtained using the murine ActRIIB inhibitor analog (RAP-031) in mouse models of muscle pathology into a nonhuman primate model and establish proof-of-principle for this drug strategy. We feel the value and benefit of the data provided were obtained in the NHP marmoset model. In addition, the muscle functional studies were performed in intact skeletal muscles using ex vivo methodology, which allows us to assess the contractile properties of the muscle itself. These types of studies are not possible in humans, which adds value to the literature. To further address this topic, we have added text to the Discussion regarding 1) muscle signaling pathways that may be involved in the ACE-031 effects on muscle and 2) the different myostatin concentrations observed across murine, NHP and humans.

We have added the following to the Discussion:

“Myostatin has been shown to induce atrophy by inhibiting signaling pathways associated with protein synthesis and activating those associated with degradation [33-36]. Whereas, inhibition of myostatin and activin A has been shown to preserve muscle mass by preventing perturbations in these pathways [33]. Activin A levels in monkeys and humans are 3-4 fold higher than mice and rats [23] and myostatin levels in mice are more than 10-fold higher than humans[24] suggesting that myostatin specific treatments that appear promising in mice are not as likely to produce clinically meaningful outcomes in humans. Indeed, although myostatin specific treatments have been shown to be efficacious in a mouse model of muscular dystrophy [2, 3, 25], selective antagonism of myostatin has failed to show meaningful clinical benefit in patients with muscular dystrophies [26-28]. Further, a systematic analysis of the pharmacokinetic and pharmacodynamic profile of a myostatin neutralizing antibody in mice, rats, monkeys and humans suggest that the amount of drug needed to induce comparable increases in muscle mass in monkeys was 20-fold higher than what is needed in mice; indicating substantial species differences in sensitivity to myostatin specific treatments[29]. Thus, the use of NHPs is likely to be a superior predictor of efficacy for treatments targeting myostatin and activin A in humans.”

We hope this rationale sufficiently addresses the Reviewer’s concern.

8) Use consistent terms for “lean mass,” “muscle mass,” and “lean body mass.”

*Response:* Thank you for this suggestion. We have changed the text to utilize “lean body mass” where appropriate.

9) State if the data were tested for normality before using parametric tests.

*Response:* This was performed. We state in the Statistical Analysis section that “Non-normally distributed variables were log-transformed so that data were normally distributed prior to statistical analysis.” We hope this sufficiently addresses the Reviewer’s concern.

10) After these revisions, the study could make a good contribution to the field.

*Response:* Thank you for your thoughtful suggestions.

References Cited in Response to Reviewers

1.	Amthor H, Macharia R, Navarrete R, Schuelke M, Brown SC, Otto A, et al. Lack of myostatin results in excessive muscle growth but impaired force generation. Proc Natl Acad Sci U S A. 2007;104(6):1835-40. Epub 2007/02/03. doi: 10.1073/pnas.0604893104. PubMed PMID: 17267614; PubMed Central PMCID: PMCPMC1794294.

2.	Bogdanovich S, Krag TO, Barton ER, Morris LD, Whittemore LA, Ahima RS, et al. Functional improvement of dystrophic muscle by myostatin blockade. Nature. 2002;420(6914):418-21. Epub 2002/12/03. doi: 10.1038/nature01154. PubMed PMID: 12459784.

3.	Bogdanovich S, Perkins KJ, Krag TO, Whittemore LA, Khurana TS. Myostatin propeptide-mediated amelioration of dystrophic pathophysiology. FASEB J. 2005;19(6):543-9. Epub 2005/03/26. doi: 10.1096/fj.04-2796com. PubMed PMID: 15791004.

4.	Bechir N, Pecchi E, Vilmen C, Le Fur Y, Amthor H, Bernard M, et al. ActRIIB blockade increases force-generating capacity and preserves energy supply in exercising mdx mouse muscle in vivo. FASEB J. 2016;30(10):3551-62. Epub 2016/07/16. doi: 10.1096/fj.201600271RR. PubMed PMID: 27416839.

5.	Carpio Y, Acosta J, Morales R, Santisteban Y, Sanchez A, Estrada MP. Regulation of body mass growth through activin type IIB receptor in teleost fish. Gen Comp Endocrinol. 2009;160(2):158-67. Epub 2008/12/06. doi: 10.1016/j.ygcen.2008.11.009. PubMed PMID: 19056390.

6.	Lee SJ, Reed LA, Davies MV, Girgenrath S, Goad ME, Tomkinson KN, et al. Regulation of muscle growth by multiple ligands signaling through activin type II receptors. Proc Natl Acad Sci U S A. 2005;102(50):18117-22. Epub 2005/12/07. doi: 10.1073/pnas.0505996102. PubMed PMID: 16330774; PubMed Central PMCID: PMCPMC1306793.

7.	Lee SJ. Quadrupling muscle mass in mice by targeting TGF-beta signaling pathways. PLoS One. 2007;2(8):e789. Epub 2007/08/30. doi: 10.1371/journal.pone.0000789. PubMed PMID: 17726519; PubMed Central PMCID: PMCPMC1949143 MetaMorphix (MMI) and sublicensed to Wyeth. The author is entitled to a share of sales royalty received by the University from sales of this factor. The Johns Hopkins University and the author also own MMI stock, which is subject to certain restrictions under University policy. The author is a paid consultant to MMI and to Merck. The terms of these arrangements are being managed by the University in accordance with its conflict of interest policies.

8.	Matsakas A, Foster K, Otto A, Macharia R, Elashry MI, Feist S, et al. Molecular, cellular and physiological investigation of myostatin propeptide-mediated muscle growth in adult mice. Neuromuscul Disord. 2009;19(7):489-99. Epub 2009/06/23. doi: 10.1016/j.nmd.2009.06.367. PubMed PMID: 19541486.

9.	McPherron AC, Lawler AM, Lee SJ. Regulation of skeletal muscle mass in mice by a new TGF-beta superfamily member. Nature. 1997;387(6628):83-90. Epub 1997/05/01. doi: 10.1038/387083a0. PubMed PMID: 9139826.

10.	McPherron AC, Lee SJ. Double muscling in cattle due to mutations in the myostatin gene. Proc Natl Acad Sci U S A. 1997;94(23):12457-61. Epub 1997/11/14. doi: 10.1073/pnas.94.23.12457. PubMed PMID: 9356471; PubMed Central PMCID: PMCPMC24998.

11.	Mosher DS, Quignon P, Bustamante CD, Sutter NB, Mellersh CS, Parker HG, et al. A mutation in the myostatin gene increases muscle mass and enhances racing performance in heterozygote dogs. PLoS Genet. 2007;3(5):e79. Epub 2007/05/29. doi: 10.1371/journal.pgen.0030079. PubMed PMID: 17530926; PubMed Central PMCID: PMCPMC1877876.

12.	Pistilli EE, Bogdanovich S, Goncalves MD, Ahima RS, Lachey J, Seehra J, et al. Targeting the activin type IIB receptor to improve muscle mass and function in the mdx mouse model of Duchenne muscular dystrophy. Am J Pathol. 2011;178(3):1287-97. Epub 2011/03/02. doi: 10.1016/j.ajpath.2010.11.071. PubMed PMID: 21356379; PubMed Central PMCID: PMCPMC3069867.

13.	Schuelke M, Wagner KR, Stolz LE, Hubner C, Riebel T, Komen W, et al. Myostatin mutation associated with gross muscle hypertrophy in a child. N Engl J Med. 2004;350(26):2682-8. Epub 2004/06/25. doi: 10.1056/NEJMoa040933. PubMed PMID: 15215484.

14.	Whittemore LA, Song K, Li X, Aghajanian J, Davies M, Girgenrath S, et al. Inhibition of myostatin in adult mice increases skeletal muscle mass and strength. Biochem Biophys Res Commun. 2003;300(4):965-71. Epub 2003/02/01. doi: 10.1016/s0006-291x(02)02953-4. PubMed PMID: 12559968.

15.	Ruiz S, Zhao H, Chandakkar P, Chatterjee PK, Papoin J, Blanc L, et al. A mouse model of hereditary hemorrhagic telangiectasia generated by transmammary-delivered immunoblocking of BMP9 and BMP10. Sci Rep. 2016;5:37366. Epub 2016/11/23. doi: 10.1038/srep37366. PubMed PMID: 27874028; PubMed Central PMCID: PMCPMC5118799.

16.	Omairi S, Matsakas A, Degens H, Kretz O, Hansson KA, Solbra AV, et al. Enhanced exercise and regenerative capacity in a mouse model that violates size constraints of oxidative muscle fibres. Elife. 2016;5. Epub 2016/08/06. doi: 10.7554/eLife.16940. PubMed PMID: 27494364; PubMed Central PMCID: PMCPMC4975572.

17.	Mouisel E, Relizani K, Mille-Hamard L, Denis R, Hourde C, Agbulut O, et al. Myostatin is a key mediator between energy metabolism and endurance capacity of skeletal muscle. Am J Physiol Regul Integr Comp Physiol. 2014;307(4):R444-54. Epub 2014/06/27. doi: 10.1152/ajpregu.00377.2013. PubMed PMID: 24965795.

18.	Kramer IF, Snijders T, Smeets JSJ, Leenders M, van Kranenburg J, den Hoed M, et al. Extensive Type II Muscle Fiber Atrophy in Elderly Female Hip Fracture Patients. J Gerontol A Biol Sci Med Sci. 2017;72(10):1369-75. Epub 2017/03/23. doi: 10.1093/gerona/glw253. PubMed PMID: 28329045.

19.	Nilwik R, Snijders T, Leenders M, Groen BB, van Kranenburg J, Verdijk LB, et al. The decline in skeletal muscle mass with aging is mainly attributed to a reduction in type II muscle fiber size. Exp Gerontol. 2013;48(5):492-8. Epub 2013/02/22. doi: 10.1016/j.exger.2013.02.012. PubMed PMID: 23425621.

20.	Tanganelli F, Meinke P, Hofmeister F, Jarmusch S, Baber L, Mehaffey S, et al. Type-2 muscle fiber atrophy is associated with sarcopenia in elderly men with hip fracture. Exp Gerontol. 2021;144:111171. Epub 2020/11/29. doi: 10.1016/j.exger.2020.111171. PubMed PMID: 33248151.

21.	Kaneguchi A, Sakitani N, Umehara T. Histological changes in skeletal muscle induced by heart failure in human patients and animal models: A scoping review. Acta Histochem. 2024;126(8):152210. Epub 2024/10/24. doi: 10.1016/j.acthis.2024.152210. PubMed PMID: 39442432.

22.	Curcio F, Testa G, Liguori I, Papillo M, Flocco V, Panicara V, et al. Sarcopenia and Heart Failure. Nutrients. 2020;12(1). Epub 2020/01/18. doi: 10.3390/nu12010211. PubMed PMID: 31947528; PubMed Central PMCID: PMCPMC7019352.

23.	Latres E, Mastaitis J, Fury W, Miloscio L, Trejos J, Pangilinan J, et al. Activin A more prominently regulates muscle mass in primates than does GDF8. Nat Commun. 2017;8:15153. Epub 2017/04/30. doi: 10.1038/ncomms15153. PubMed PMID: 28452368; PubMed Central PMCID: PMCPMC5414365 Pharmaceuticals. E.L. and T.W. were employees of Regeneron Pharmaceuticals when this study was conducted. J.M., W.F., L.M., J.T., J.P., H.O., K.C., E.N., A.P., Y.B., J.H.K., A.R., S.J., T.S., A.J.M., G.D.Y. and J.G. are currently employees and hold stock or stock options of Regeneron Pharmaceuticals. Regeneron Pharmaceuticals and E.L., T.S., A.J.M., G.D.Y. and J.G. hold patents for antibodies inhibiting GDF8 and activin A and uses thereof.

24.	Lakshman KM, Bhasin S, Corcoran C, Collins-Racie LA, Tchistiakova L, Forlow SB, et al. Measurement of myostatin concentrations in human serum: Circulating concentrations in young and older men and effects of testosterone administration. Mol Cell Endocrinol. 2009;302(1):26-32. Epub 2009/04/10. doi: 10.1016/j.mce.2008.12.019. PubMed PMID: 19356623.

25.	St Andre M, Johnson M, Bansal PN, Wellen J, Robertson A, Opsahl A, et al. A mouse anti-myostatin antibody increases muscle mass and improves muscle strength and contractility in the mdx mouse model of Duchenne muscular dystrophy and its humanized equivalent, domagrozumab (PF-06252616), increases muscle volume in cynomolgus monkeys. Skelet Muscle. 2017;7(1):25. Epub 2017/11/11. doi: 10.1186/s13395-017-0141-y. PubMed PMID: 29121992; PubMed Central PMCID: PMCPMC5679155.

26.	Wagner KR, Fleckenstein JL, Amato AA, Barohn RJ, Bushby K, Escolar DM, et al. A phase I/IItrial of MYO-029 in adult subjects with muscular dystrophy. Ann Neurol. 2008;63(5):561-71. Epub 2008/03/13. doi: 10.1002/ana.21338. PubMed PMID: 18335515.

27.	Wagner KR, Abdel-Hamid HZ, Mah JK, Campbell C, Guglieri M, Muntoni F, et al. Randomized phase 2 trial and open-label extension of domagrozumab in Duchenne muscular dystrophy. Neuromuscul Disord. 2020;30(6):492-502. Epub 2020/06/12. doi: 10.1016/j.nmd.2020.05.002. PubMed PMID: 32522498.

28.	Muntoni F, Byrne BJ, McMillan HJ, Ryan MM, Wong BL, Dukart J, et al. The Clinical Development of Taldefgrobep Alfa: An Anti-Myostatin Adnectin for the Treatment of Duchenne Muscular Dystrophy. Neurol Ther. 2024;13(1):183-219. Epub 2024/01/08. doi: 10.1007/s40120-023-00570-w. PubMed PMID: 38190001; PubMed Central PMCID: PMCPMC10787703.

29.	Singh P, Rong H, Gordi T, Bosley J, Bhattacharya I. Translational Pharmacokinetic/Pharmacodynamic Analysis of MYO-029 Antibody for Muscular Dystrophy. Clin Transl Sci. 2016;9(6):302-10. Epub 2016/10/05. doi: 10.1111/cts.12420. PubMed PMID: 27700008; PubMed Central PMCID: PMCPMC5351001.

30.	Pistilli EE, Bogdanovich S, Mosqueira M, Lachey J, Seehra J, Khurana TS. Pretreatment with a soluble activin type IIB receptor/Fc fusion protein improves hypoxia-induced muscle dysfunction. Am J Physiol Regul Integr Comp Physiol. 2010;298(1):R96-R103. Epub 2009/10/30. doi: 10.1152/ajpregu.00138.2009. PubMed PMID: 19864340; PubMed Central PMCID: PMCPMC2806202.

31.	O'Connell G, Guo G, Stricker J, Quinn LS, Ma A, Pistilli EE. Muscle-specific deletion of exons 2 and 3 of the IL15RA gene in mice: effects on contractile properties of fast and slow muscles. J Appl Physiol (1985). 2015;118(4):437-48. Epub 2014/12/17. doi: 10.1152/japplphysiol.00704.2014. PubMed PMID: 25505029.

32.	Pistilli EE, Bogdanovich S, Garton F, Yang N, Gulbin JP, Conner JD, et al. Loss of IL-15 receptor alpha alters the endurance, fatigability, and metabolic characteristics of mouse fast skeletal muscles. J Clin Invest. 2011;121(8):3120-32. Epub 2011/07/19. doi: 10.1172/JCI44945. PubMed PMID: 21765213; PubMed Central PMCID: PMCPMC3148729.

33.	Zhou X, Wang JL, Lu J, Song Y, Kwak KS, Jiao Q, et al. Reversal of cancer cachexia and muscle wasting by ActRIIB antagonism leads to prolonged survival. Cell. 2010;142(4):531-43. Epub 2010/08/21. doi: 10.1016/j.cell.2010.07.011. PubMed PMID: 20723755.

34.	Trendelenburg AU, Meyer A, Rohner D, Boyle J, Hatakeyama S, Glass DJ. Myostatin reduces Akt/TORC1/p70S6K signaling, inhibiting myoblast differentiation and myotube size. Am J Physiol Cell Physiol. 2009;296(6):C1258-70. Epub 2009/04/10. doi: 10.1152/ajpcell.00105.2009. PubMed PMID: 19357233.

35.	Welle S, Burgess K, Mehta S. Stimulation of skeletal muscle myofibrillar protein synthesis, p70 S6 kinase phosphorylation, and ribosomal protein S6 phosphorylation by inhibition of myostatin in mature mice. Am J Physiol Endocrinol Metab. 2009;296(3):E567-72. Epub 2009/01/15. doi: 10.1152/ajpendo.90862.2008. PubMed PMID: 19141683; PubMed Central PMCID: PMCPMC2660146.

36.	McFarlane C, Plummer E, Thomas M, Hennebry A, Ashby M, Ling N, et al. Myostatin induces cachexia by activating the ubiquitin proteolytic system through an NF-kappaB-independent, FoxO1-dependent mechanism. J Cell Physiol. 2006;209(2):501-14. Epub 2006/08/03. doi: 10.1002/jcp.20757. PubMed PMID: 16883577.
